# Detection of the Janus kinase 2 V617F mutation using a locked nucleic-acid, real-time polymerase chain reaction assay

**DOI:** 10.4102/ajlm.v7i1.658

**Published:** 2018-01-31

**Authors:** Tshiphiri Senamela, Marleen Kock, Piet Becker, Joachim J.C. Potgieter

**Affiliations:** 1 National Health Laboratory Services, Pretoria, South Africa; 2 Department of Haematology, Faculty of Health Sciences, University of Pretoria, Pretoria, South Africa

## Abstract

The purpose of this study was to develop a real time polymerase chain reaction (PCR) assay for the detection of the *JAK2* V617F mutation that could be used in diagnostic laboratories. Sanger sequencing and a newly developed locked nucleic-acid, real-time PCR assay were used to detect the *JAK2* V617F mutation. There was 100% agreement between the sequencing and PCR analysis. Both assays were able to detect the mutation in all 24 of the 60 test specimens harbouring the mutation.

## Introduction

Myeloproliferative neoplasms (MPNs) are a heterogeneous group of clonal disorders that result from mutational transformation of a haematopoietic stem cell.^1^ This transformation of haematopoietic stem cells causes uncontrolled proliferation of myeloid cell lines leading to overproduction of both mature and immature blood cells.^1^

Prior to 2005, no underlying genetic abnormality had been associated with classic breakpoint cluster region-Abelson leukaemia virus negative MPNs. In 2005, a mutation at base 1849 in exon 14 of the *JAK2* gene on chromosome 9 was discovered in patients with polycythaema vera, essential thrombocythaemia and primary myelofibrosis.^2,3,4^ This somatic point mutation causes a substitution of guanine by thymine and the amino acid is changed from valine to phenylalanine in codon 617 of the JAK2 protein.^2^ This discovery has improved understanding of the pathophysiology of MPNs and has renewed interest and research in MPN biology and genetics.^5,6^

The ability to demonstrate the presence of this mutation has not only simplified the diagnosis of MPN but also ensured greater diagnostic accuracy.^5^ The identification of this mutation has also led to the development of therapies targeted at inhibition of the JAK2 kinase.^7,8^

Sensitive and specific assays are required for the detection of the *JAK2* V617F mutation.^9^ Molecular assays with high sensitivity and specificity should be offered by diagnostic laboratories for this purpose.^9^ Several different commercial and in-house assays that offer different sensitivity and specificity levels have been developed.^9^ Direct sequencing is a gold standard for mutation analysis but is limited by low sensitivity and high cost.^10^

In-house assays that are currently used include allele-specific polymerase chain reaction or amplification refractory mutation system, direct sequencing, polymerase chain reaction (PCR) with restriction fragment length polymorphism (PCR-RFLP) and real-time PCR.^9,11,12^ Diagnostic laboratories may find it challenging to select the most appropriate methodology to detect the *JAK2* mutation.^13^ However, the use of a reliable, quick and sensitive assay that is able to detect 1% of the mutant allele in a wild-type background is recommended.^13^ The aim of this study was to develop a real-time PCR assay for the detection of the *JAK2* V617F mutation for implementation in a routine clinical laboratory.

## Methods

### Ethical considerations

The study received approval from the University of Pretoria Faculty of Health Sciences Research Ethics Committee (S32/2012) for using sample remnants for further analysis. Leftover blood samples were collected anonymously from a Tshwane tertiary hospital for DNA extraction and PCR assay. All samples were used solely for the detection of the *JAK2* V617F mutation.

### Samples collected and study site

This study used specimens of whole blood collected in EDTA (*n* = 60) that were submitted by clinicians from Steve Biko Academic Hospital to be evaluated for the presence of the *JAK2* V617F mutation in patients suspected to have MPNs. The study was conducted between 01 October 2013 and 30 September 2015.

### Genomic DNA

Genomic DNA (gDNA) was extracted from 0.2 mL of peripheral blood using the GenElute™ blood genomic DNA kit (Sigma Aldrich, St. Louis, Missouri, United States) according to the manufacturer’s instructions. The concentration of DNA was confirmed by the NanoDrop 2000c UV spectrophotometer (Thermo Scientific, Waltham, Massachusetts, United States) to obtain gDNA with a concentration of 1.6 to 1.9 µg/mL. Extracted DNA was stored at –20°C until analysis.

### Sequencing of amplified products

Primers for the detection of the single nucleotide polymorphism in codon 617 were designed based on the known DNA sequence of the *JAK2* gene (Genbank^®^ accession number NG_009904.1) using the PrimerQuest software (Integrated DNA Technologies, Coralville, Iowa, United States) (Table 1). The product of interest was amplified using the DNA Engine Peltier thermocycler (Bio Rad, München, Germany) under the following conditions: initial denaturation at 94°C for 10 minutes, followed by 35 cycles of amplification with denaturation at 94°C for 30 seconds, annealing at 52°C for 30 seconds and extension at 72°C for 45 seconds. Sanger sequencing was performed by Inqaba Biotechnical Industries (Pretoria, South Africa) in both directions on all 60 amplified products. The CLC main workbench software program v6.0 (CLCBio, Waltham, Massachusetts, United States) was used to analyse the sequences against the NCBI reference sequence NG_009904.

**TABLE 1:** Polymerase chain reaction primer and probe sequences used to detect the *JAK2* V617F mutation (5′ → 3).

Forward primer	AGGGACCAAAGCACATTGTAT
Reverse primer	CCTAGCTGTGATCCTGAAACTG
Wild-type probe	HEX GATG^†^T^†^G^†^T^†^CT^†^G^†^TGG Lower black FQ
Mutant probe	FAM ATG^+^T^+^T^+^T^+^CT^+^GT^+^G^+^GAG Lower black FQ

LNA, locked nucleic acid.

† Indicates LNA bases

### Locked nucleic-acid, real-time polymerase chain reaction assay

Wild-type and mutant probes were designed to be complementary to the wild-type and mutant nucleotide sequences (Table 1). The real-time PCR assay was performed using the Cepheid SmartCycler II platform (Cepheid, Maurens-Scopont, France). The reaction mixture contained 0.4 µM each of the forward and reverse primers, 0.4 µM of mutant and 0.05 µM of wild-type probes, 2X Qiagen QuantiNova^TM^ probe PCR master mix (Qiagen, Hilden, Germany) and < 100 ng template gDNA. In an attempt to optimise the assay’s performance, the wild-type probe concentration was adjusted in a separate experiment by making a serial dilution of the wild-type probe in nuclease-free water and assessing its performance. The concentration of the wild-type probe that gave optimal results was found to be 0.05 µM. This concentration of wild-type probe was used together with 0.4 µM mutant probe in the PCR assay with satisfactory results. Polymerase chain reaction was performed under the following conditions: initial denaturation at 95°C for two minutes, followed by 40 cycles of amplification with denaturation at 95°C for 10 seconds, then combined annealing and extension at 60°C for 30 seconds. Genomic gBlocks^®^ Gene Fragments (Integrated DNA Technologies, Coralville, Iowa, United States) consisting of mutant and wild-type sequences each together with no template control were included with each run.

The analytical sensitivity of the locked nucleic-acid (LNA) real-time PCR assay was determined using wild-type DNA mixed with that of homozygous mutant DNA in various concentrations (100% mutant, 50% mutant, 20% mutant, 10% mutant, 5% mutant, 2% mutant, 1% mutant and 0.1% mutant). The dilutions were prepared fresh and subjected to PCR in three replicate experiments to ensure reproducibility of the assay. The limit of detection was defined as the lowest dilution of mutant DNA in which all three replicates resulted in positive amplification.^14^

### Data and statistical analysis

The real-time PCR assay results were compared to the sequencing assay results to assess the assay’s usefulness for allelic discrimination of the *JAK2* V617F mutation. The results were used to assess the agreement between the two assays using Cohen’s Kappa for inter-rater agreement to assess the agreement between methods using interpretation cut-offs: κ > 0.75 excellent agreement, κ = 0.4–0.75 good agreement and κ < 0.4 poor agreement.^15^

## Results

**FIGURE 1:**
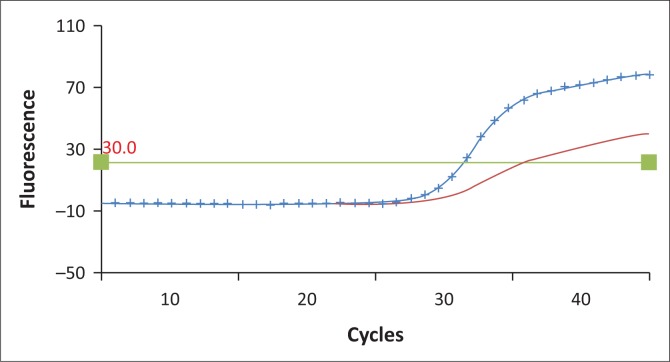
An amplification curve showing results with 0.4 µM wild-type and 0.4 µM mutant probes. Mutated allele curve with no markers, wild-type allele curve with crosses.

**FIGURE 2:**
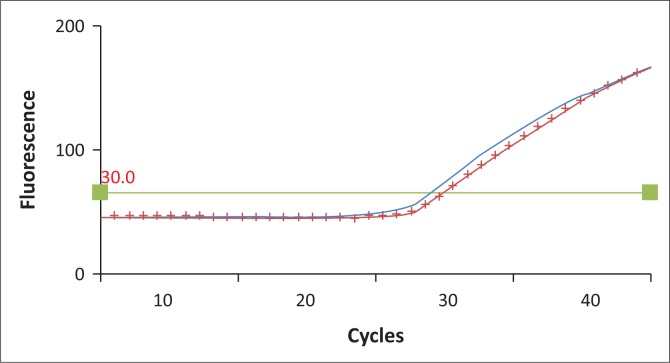
An amplification curve showing results with 0.05 µM wild-type and 0.4 µM mutant probes. Mutated allele curve with no markers, wild-type allele curve with crosses.

**FIGURE 3:**
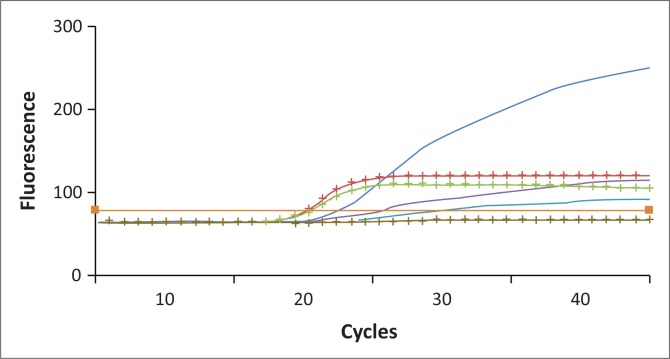
Amplification curves showing the analytical sensitivity of one sample. Green: 100% homozygous mutant DNA; red: 20% mutant with 80% wild-type DNA; blue: 0.1% mutant DNA in 99.9% of wild-type DNA.

Both sequencing and the LNA real-time PCR assay detected the mutation in the same 24 samples (40%) and with κ = 1 (i.e. perfect agreement). During the experiment, it was noted that the standard 1:1 wild-type to mutant probe ratio did not provide optimal results. The ratio of wild-type to mutant probe that provided optimal results was 1:8 (Figures 1 and 2 show how a heterozygous sample amplifies when the 1:1 and 1:8 ratios of mutant to wild-type probes were used). The limit of detection of this assay is 0.1% in a wild-type DNA background (Figure 3).

## Discussion

In this study, an LNA probe-based, real-time PCR assay was developed and evaluated for its ability to detect the *JAK2* V617F mutation. An LNA is an analogue of nucleotides that contains an internal 2′-O, 4′-C methylene bridge, which locks the ribose ring into a C3′-endo conformation.^16^ Introduction of LNAs into probes increases the thermal stability of the probe by + 3°C to + 8°C per modification and allows binding to complementary target sequences with high affinity.^16,17,18^ In this study, the wild-type probe contained six LNAs and the mutant probe seven LNAs. Increasing the number of LNAs in the mutant probe was necessary to increase the melting temperature (T_m_) of the probe to greater than that of the primers and thus increase the specificity of the probe. The wild-type probe binds with high affinity, thus reducing the intensity of the mutant probe as it is competing with the mutant probe in this multiplexed assay. Both sequencing and LNA real-time PCR assays detected the mutation in 40% of the samples. The agreement between the real-time PCR assay and sequencing was 100%.

Consistent positive amplification was obtained down to the 0.1% dilution (Figure 3). The limit of detection is determined experimentally by preparing serial dilutions of mutant DNA in a wild-type DNA background and analysing each dilution point in a six-fold run.^14^ The last dilution where all six replicates give a positive and specific amplification is considered to be the limit of detection.^14^ The acceptable sensitivity of a qualitative test should be equal to or below 20%.^14^ However, it is recommended that assays for the detection of the *JAK2* V617F mutation in a clinical setting should have an analytical sensitivity of at least 1% to ensure that more than 90% of cases are detected.^13^ Our newly designed LNA probe, real-time PCR assay detects up to 0.1% of mutant DNA in a wild-type DNA background using gDNA in contrast to an earlier assay with an analytical sensitivity of 2% using complementary DNA.^19^ This may be an improvement on previously published probes.

With the good analytical sensitivity of this assay, it is considered suitable for use in a diagnostic laboratory and constitutes a good screening tool. However, it should be noted that an assay with this sensitivity may be prone to give more false positives in a diagnostic setting.^13^

### Limitations

To assess the robustness of a real-time PCR assay different experimental conditions, such as annealing temperatures, the use of different instruments and operators should be introduced.^14^ This aspect of assay evaluation was not explored in this study. However, to show that the assay was able to perform equally well on another platform, the DNA samples were analysed on the Qiagen Rotor-Gene Q 2Plex system (Germany). Similar results were obtained to that seen using the Cepheid SmartCycler II system.

### Conclusion

The developed LNA probe, real-time PCR assay is a suitable diagnostic method for detecting the *JAK2* V617F mutation in a clinical setting. It has good sensitivity, is easy to set up and has rapid turn-around times.
